# Utility of Logistic Regression Analysis to Estimate Prognosis in Acute Myocardial Infarction

**DOI:** 10.4103/0970-0218.58408

**Published:** 2009-10

**Authors:** Frederick S Vaz, AM Ferreira, MS Kulkarni, DD Motghare

**Affiliations:** Department of Preventive and Social Medicine, Goa Medical College, Bambolim, Goa – 403 202, India. E-mail: frederickvaz@rediffmail.com

Sir,

In their award winning research paper published in IJCM, Kakade *et al.*(1) have successfully demonstrated the application of logistic regression techniques in identifying the predictors of prognosis in patients with acute myocardial infarction (AMI). We conducted a study at a tertiary care Hospital in Goa using identical methodology^1^ with the aim of replicating the study in a different study setting.

About 321 consecutive patients with AMI admitted at the hospital during 2007 were studied. The study sample included 258 (80.4%) males and 63 (19.6%) females. Around 59.8% of patients (192/321) were in the 50-70 years age group, 63 patients (19.6%) were under 50 years, and 66 patients (20.6%) were over 70 years old. There were 68 deaths (21.2%) during treatment among the AMI inpatients and there was no significant difference in mortality rates among males and females (28% *vs* 19.4%; *P*=0.10).

In our study, binary logistic regression analysis identified only three predictor variables for the prognosis of AMI inpatients [Table 1]. Patients reporting early to the hospital, having longer stay and those with higher systolic blood pressure at admission, were likely to have better prognosis during their hospital stay. Length of hospital stay operated differently than other predictors, that is, if a patient survives the first 48 h, then his risk of dying from AMI decreases significantly with further increase in the length of stay.

**Table 1:** Significant determinants of prognosis in AMI in-patients

Variable	No. *N* = 321	Survived number (%) *N* = 253	Died number (%) *N* = 68	Unadjusted OR (95% CI)	Adjusted OR (95% CI)
Time gap in treatment					
0-6 h	146	124 (84.9)	22 (15.1)	1 (Ref)	1 (Ref)
6-12 h	44	34 (77.3)	10 (22.7)	1.66 (0.66-4.12)	2.37 (0.69-8.09)
12-24 h	18	18 (100)	0 (0)	0.00*	0.00*
24+ h	87	59 (67.8)	28 (32.2)	2.67 (1.35-5.32)	4.27 (1.63-11.17)
NA	26	18 (69.2)	8 (30.8)	-	-
Hospital stay					
<48 h	47	7 (14.9)	40 (85.1)	63.67 (26.3-179.2)	107.96 (31.37-371.60)
48-96 h	19	12 (63.2)	7 (36.8)	6.50 (2.05-20.33)	11.66 (3.05-44.52)
96+ h	255	234 (91.8)	21 (8.2)	1 (Ref)	1 (Ref)
Systolic BP					
≥ 140 mmHg	126	115 (91.3)	11 (8.7)	1 (Ref)	1 (Ref)
<140 mmHg	193	137 (71.0)	56 (29.0)	4.27 (2.05-9.09)	5.39 (1.92-15.17)
NA	2	1 (50)	1 (50)	-	-

* No deaths in this category, results inconsistent, NA: Data not available, Adjusted odds ratio obtained by Binary Logistic regression analysis

AMI patients reporting after more than 24 h of onset were 4.27 times more likely to die during hospital stay compared to those reporting within 6 h of onset of AMI. Those having ‘at admission’ systolic blood pressure of less than 140 mmHg were 5.39 times more likely to die than those with at admission blood pressure measurement of more than 140 mmHg.

Kakade *et al.*(1) found that on logistic regression analysis; age, gender, place of residence, time gap in treatment, and hospital treatment were the significant variables. Jiang *et al.*(2) using a multivariate logistic regression model, identified age, history of hypertension, and diabetes mellitus as significant predictors of in-hospital mortality in patients with AMI. Yap *et al.*(3) reported that low systolic blood pressure was a significant predictor of mortality in AMI patients. Ivanusa *et al.*(4) also reported that survivors of AMI had higher prevalence of hypertension.

Although the predictors identified by our analysis are slightly different from those identified by other studies,(1,2) they could be of immense use to physicians treating AMI patients. Patient outcomes in acute myocardial infarction could be improved by the consistent use of these predictors as they would alert the physician about the potential bad outcomes resulting in institution of timely interventions in identified ‘at risk of dying’ patients.
